# CircFOXM1 promotes proliferation and metastasis of hepatocellular carcinoma via regulating miR-1179/SPAG5 axis

**DOI:** 10.1038/s41598-021-03285-w

**Published:** 2021-12-13

**Authors:** Gaoqing Wang, Yin Jiang, Caide Lu, Wei Jiang, Shugeng Wu, Yongfei Hua

**Affiliations:** Department of Hepatopancreatobiliary Surgery, Ningbo Medical Center Lihuili Hospital, Ningbo University, Ningbo, Zhejiang 315040 China

**Keywords:** Cancer, Cancer genetics, Cancer genomics

## Abstract

Hepatocellular carcinoma (HCC) predominantly occurs in patients with chronic liver disease, accounting for 70–90% of all liver cancer cases. The role of circFOXM1/miR-1179/SPAG5 axis in HCC has not been reported. This study aimed to explore the regulatory mechanism of circFOXM1 in HCC proliferation and metastasis. RNA polymerase inhibitor actinomycin D and RNase R exonuclease were used to identify circFOXM1 in HCC cells. The qRT-PCR was used to detect circFOXM1 expression. Specific siRNA for circFOXM1 was designed, and the sequence of circFOXM1 was inserted in pLCDH-ciR to overexpress circFOXM. Cell proliferation was detected by CCK8 in vitro*,* by tumor volume and tumor weight of HCC xenograft in vivo. Cell migration was detected by transwell test. Binding status of circFOXM1 with miR-1179 was detected by luciferase reporter gene assay. Rescue experiments were applied to identify the oncogenic mechanism of circFOXM1 in HCC cells. Actinomycin D assay confirmed the cyclization of circFOXM1. RNase R treatment showed that circFOXM1 was not affected by RNase R exonuclease. CCK8 assay, tumor volume and tumor weight showed that circFOXM1 effectively promoted HCC cell proliferation. Transwell assay showed that circFOXM effectively promoted migration and invasion abilities of HCC cells. Luciferase reporter gene activity assay showed that miR-1179 had complementary binding sites with circFOXM1 and SPAG5. CircFOXM1 silencing inhibited malignant phenotypes in HCC cells were partly rescued by either miR-1179 silencing or SPAG5 overexpression. CircFOXM1 promoted HCC cell proliferation and metastasis by regulating miR-1179/SPAG5 axis.

## Introduction

Hepatocellular carcinoma (HCC) is the most common liver cancer, accounting for about 70–90%. HCC rates the second cause of cancer-related death with 750,000 deaths every year in the world^1^. Though the diagnosis and treatment strategies of HCC have made great progress, the prognosis of HCC is still poor appearing a high risk of metastasis^2^, therefore, it is of great significance to study the molecular mechanism of hepatocarcinogenesis for identifying new therapeutic strategies and potential biomarkers.

Cyclic ribosomal RNA is a biological messenger widely presenting in eukaryotes and a type of endogenous non-coding RNA with a closed covalent loop^3^. Studies have suggested that circRNAs are more resistant to RNA exonuclease degradation and mainly in cytoplasm^4^. In recent years, with the development of bioinformatics and biomolecular technologies, circRNAs have been confirmed to participate in many biological processes, and play biological functions by directly regulating miRNAs^5,6^. Meanwhile, circRNAs play important roles in tumorigenesis and development^7^. For example, circCCDC9 regulates CAV1 expression by directly targeting miR-6792-3p cavernous body, thereby inhibiting the development of gastric cancer^8^; circTADA2A plays a role in the biological progress of osteosarcoma through the miR-203a-3p/CREB3 axis^9^; circRNA FOXM1 has also been reported to sponge miR-1179, thereby accelerating the progression of papillary thyroid carcinoma^10^. However, the biological role and mechanism of FOXM1 in HCC has not been fully elucidated.

MicroRNAs (miRNAs) are the mostly studied non-coding RNAs with about 20–24 nucleotides in length. The main way of miRNAs to play biological roles is binding to the 3′UTR of mRNAs, thereby inhibiting mRNA expressions^11^. Studies have shown that miRNAs play important roles in occurrence and development of many diseases, including proliferation, invasion and apoptosis of cancer cells^12,13^. Meanwhile, studies have also confirmed that circRNAs regulate the occurrence and development of tumors by binding to miRNA sponges^14^. For example, circRNA_100290 sponging miR-29 plays an important role in the biological progression of oral squamous cell carcinoma^15^. Moreover, miR-1179 has been reported to inhibit HCC metastasis by interacting with ZEB2^16^. By applying the circRNA interactors (https://circintercome.nia.nih.gov/), the potential circRNA/miRNA interactions were predicted, and miR-1179 was identified to be the first one in the miRNA list harboring binding sites with the circFOXM1 (hsa_circ_0025033) sequence. Furthermore, the important regulatory role of the circFOXM1-miR-1179 network has not yet been elucidated in HCC development.

In this study, we revealed that circFOXM1 expression was significantly upregulated in HCC cells and tissues; the circFOXM1/miR-1179/SPAG5 axis played an important regulatory role in the biological process of HCC, which provided a theoretical basis for the diagnosis and treatment of HCC.

## Materials and methods

All methods used in this study were performed in accordance with the relevant guidelines and regulations.

### Cell culture and transfection

Human HCC cell lines (Huh7, HepG2, Hep3B, HCCLM3 and MHCC97-H) and normal human hepatocytes (MIHA) were obtained from Shanghai Institute of Cell Biology (Shanghai, China). Cells were maintained in DMEM or RPMI-1640 medium (HyClone) containing 10% fetal bovine serum (FBS) under the condition of 5% CO_2_ and 37 °C.

The specific circFOXM1 or SPAG5 siRNA was designed and synthesized by GenePharma (Shanghai, China). The sequence of circFOXM1 or SPAG5 was inserted in pLCDH-ciR to construct the recombinant overexpression vector respectively. HCC cells were transfected with circFOXM1 or SPAG5 siRNA, pLCDH-circFOXM1 or pLCDH-SPAG5, miR-1179 mimics or miR-1179 inhibitor using Lipofectamine 2000 (Invitrogen), following the manufacturer’s instructions.

### Actinomycin D assay

Cells were exposed to 2 mg/ml actinomycin D (Sigma) for transcription inhibition. And the RNA stabilities of circFOXM1 and FOXM1 were analyzed by qRT-PCR.

### RNase R treatment

Incubated 10 μg total RNA with 3 U/μg RNase R (Epicentre Technologies) at 37 °C for 15 min, and the RNA stabilities of circFOXM1 and FOXM1 were analyzed by qRT-PCR.

### qRT-PCR

Total RNAs were extracted using TRIzol reagent (Invitrogen). qRT-PCR for mRNA detection was performed with SYBR Premix Ex Taq (Takara) system. qRT-PCR for miRNA detection was performed with SYBR Prime Script miRNA RT-PCR kit (Takara). U6 was used as an internal control for miRNA; GAPDH was used as an internal control for circRNA and mRNA. ABI 7500 real-time PCR system (Life Technology Corporation) was used for qRT-PCR reaction. The relative gene expression was calculated by 2^−∆∆CT^. The relative expressions of circRNA and mRNA were normalized to GAPDH. The relative miRNA expression was normalized to human U6.

### CCK-8 proliferation assay

A 10 μL of CCK-8 reagent (Dojindo, Japan) was added into a 96-well plate with 3000 cells per well, and incubated at 37 °C for 2 h. The OD was measured at 450 nm with a microplate reader (Thermo).

### Transwell analysis

Cells (0.5 × 10^5^) were suspended in 200 μl medium without FBS and seeded into the upper compartments of “Transwell” chambers (BD Biosciences, Heidelberg, Germany). The medium containing 10% FBS was injected into the bottom chamber, and the cells were stained with crystal violet for 15 min. After 24 h incubation, cells migrated to the lower surface were fixed and stained. For cell invasion assay, Matrigel (Sigma) was plated into the upper chamber surfaces.

### Luciferase reporter gene detection

The pGL3 luciferase reporter vector (Promega) of miR-1179 mimic or NC mimic was prepared, the circFOXM1-WT or circFOXM1-MUT sequence was constructed, and then co-transfected into cells using the Lipofectamine 2000. The luciferase activity was measured after 24 h of co-cultivation and normalized to the internal control (Renilla luciferase).

### Western blot assay

Cell lysates were extracted using RIPA buffers (Beyotime Biotechnology, Shanghai, China) and boiled at 100 °C for 5 min. Then, the proteins were transferred to polyvinylidene fluoride (PVDF) membrane, blocked using non-fat dried milk, incubated with primary antibodies at 4 °C overnight, washed and probed with horseradish peroxidase (HRP)-conjugated secondary antibody, and visualized with ECL Plus chemiluminescence reagent (Beyotime Biotechnology).

### Xenograft experiments

Ten female 5-week-old BALB/c nude mice were provided by Beijing HFK Bioscience Co. Ltd. (Beijing, China), and divided into 2 groups for HCC xenograft model preparation. Then the Huh7 cells with or without circFOXM1 silencing in 200 μl PBS were respectively injected subcutaneously into flank of each mouse (1 × 10^7^ cells per mouse). The minimum (W) and maximum (L) length of tumors were measured using a vernier caliper every week to calculate the tumor volume. The mice were sacrificed by cervical dislocation following inhaling CO_2_, after 4 weeks to isolate and weight the tumors. All animal experiments were accomplished following the ethical regulations approved by the Biomedical Ethics Committee of Li HuiLi Hospital.

### Statistical analysis

SPSS 24.0 (IBM, Chicago, USA) was used to perform statistical analysis on the experimental results obtained in this research. The data differences between the groups were analyzed by Student’s *t* test and one-way analysis. *p* < 0.05 was used as a statistically significant standard.

### Ethical approval

This study has been reported in accordance with ARRIVE guidelines.

## Results

### Identification of circFOXM1 in HCC cells

**Figure 1:**
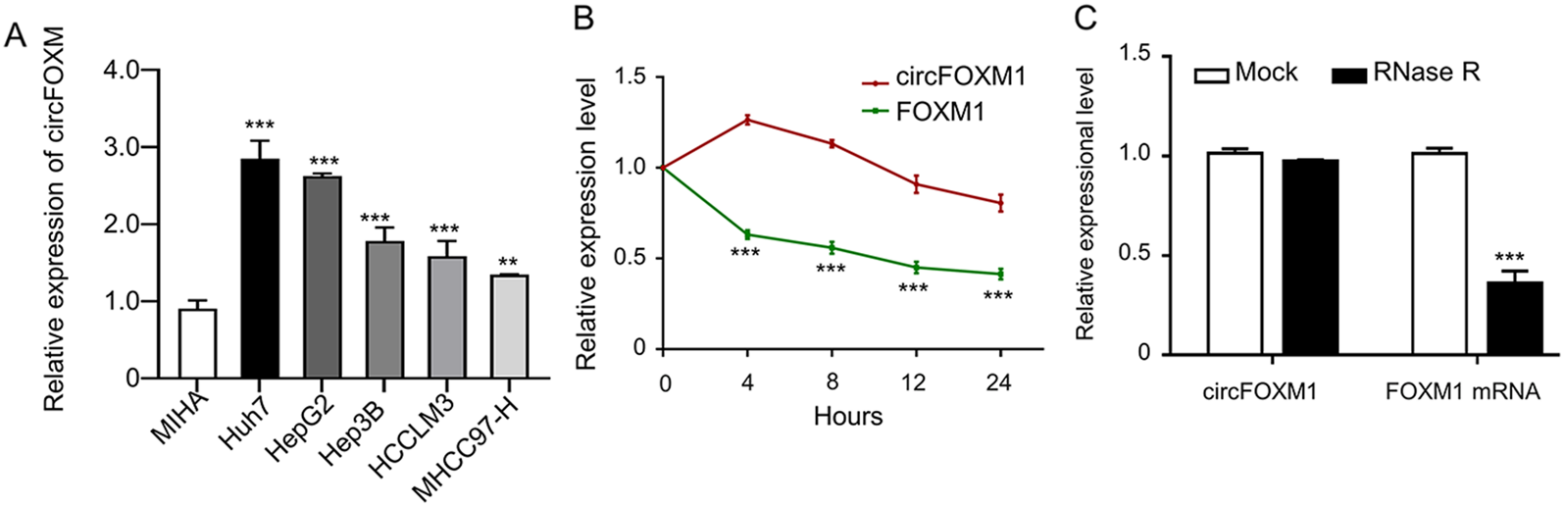
The expression level and stability of circFOXM1 in HCC cells detected by qRT-PCR. (**A**) circFOXM1 (circBase: hsa_circ_0025033) expression levels in different HCC cells; (**B**) mRNA expression abundance of circFOXM1 and FOXM1 in Huh7 cells treated with actinomycin D at the specified time points; (**C**) RNA expressions of FOXM1 and circFOXM1 in Huh7 cells with or without RNase R treatment.

To identify the expression status of circFOXM1 in HCC cells, circFOXM1 expression levels in different HCC cells (Huh7, HepG2, Hep3B, HCCLM3 and MHCC97-H) and the normal human hepatocytes (MIHA) were compared, which showed that circFOXM1 expressions in Huh7, HepG2, Hep3B, HCCLM3 and MHCC97-H cells were all significantly upregulated versus MIHA, with the highest expression level in Huh7 cells (Fig. 1A). Herein, the stability of circFOXM1 in HCC cells was further investigated using Huh7 cells. After both the circFOXM1 and FOXM1 in Huh7 cells were exposed to Actinomycin D (Fig. 1B) or Ribonuclease R (RNase R, Fig. 1C), the expressions of circFOXM1 and FOXM1 were detected by qRT-PCR respectively, which revealed that circFOXM1 was more stable and more resistant to RNase R digestion than FOXM1.

### CircFOXM1 promotes proliferation and metastasis of HCC cells in vitro, and HCC xenograft growth in vivo

**Figure 2:**
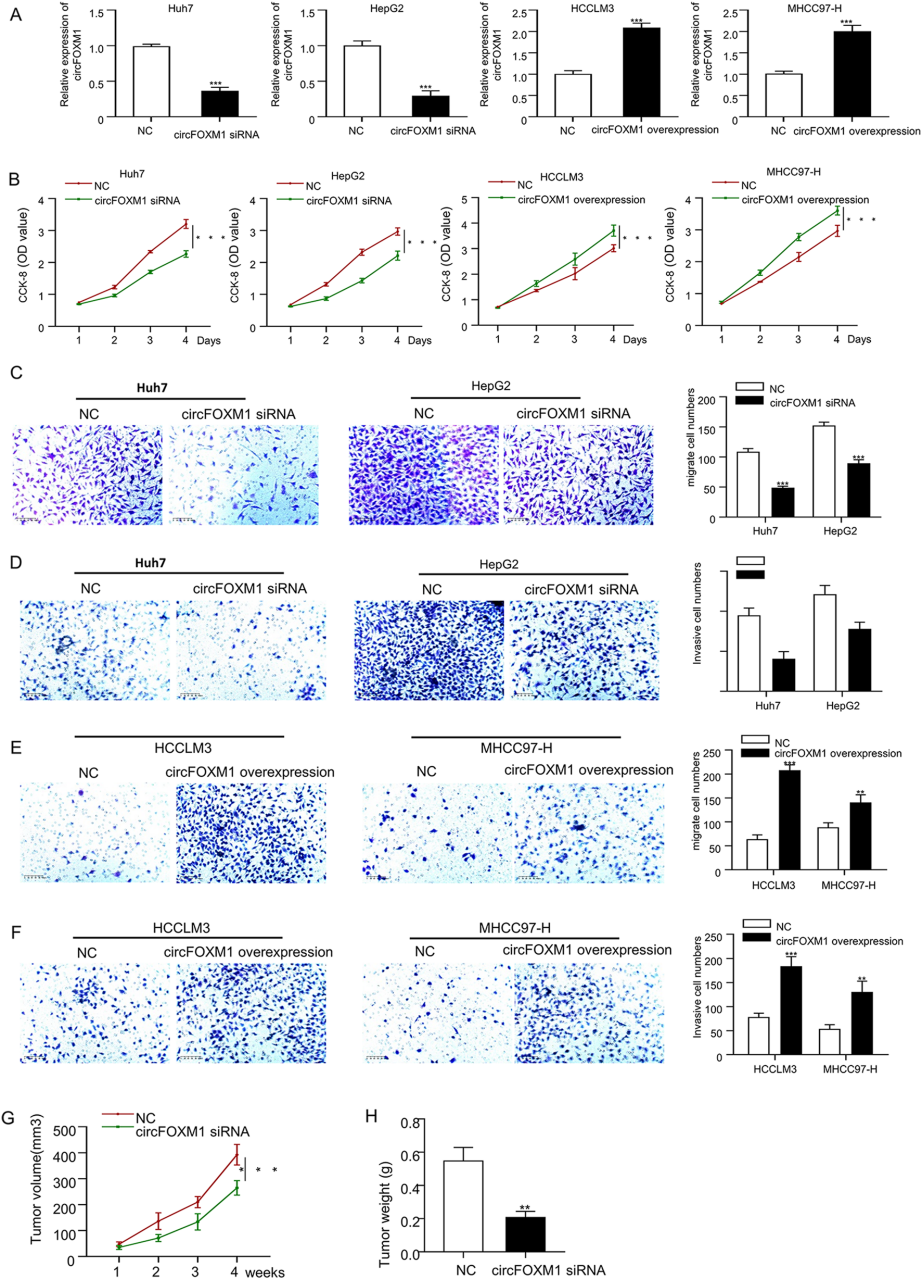
CircFOXM1 promotes proliferative and metastatic abilities of HCC. (**A**) The circFOXM1 knockdown efficacy after transfection of circFOXM1 siRNA in Huh7 and HepG2 cells, as well as the circFOXM1 overexpression efficacy after transfection of pLCDH-circFOXM1 in HCCLM3 and MHCC97-H cells. (**B**) Viabilities determined by CCK-8 assay in Huh7 and HepG2 HCC cells transfected with NC or circFOXM1 siRNA, and in HCCLM3 and MHCC97-H HCC cells transfected with NC or pLCDH-circFOXM1. (**C**) Migration ability determined by transwell assay in Huh7 and HepG2 HCC cells transfected with NC or circFOXM1 siRNA. (**D**) Invasion ability determined by transwell assay in Huh7 and HepG2 HCC cells transfected with NC or circFOXM1 siRNA. (**E**) Migration ability determined by transwell assay in HCCLM3 and MHCC97-H HCC cells transfected with NC or pLCDH-circFOXM1. (**F**) Invasion ability determined by transwell assay in HCCLM3 and MHCC97-H cells transfected with NC or pLCDH-circFOXM1. CircFOXM1 silencing inhibits HCC xenograft growth in vivo (n = 5). (**G**) Tumor volume, (H) Tumor weight. *p < 0.05; **p < 0.01, ***p < 0.001.

To explore the biological function of circFOXM1 in HCC cells, circFOXM1 was either silenced by transfection of circFOXM1 specific siRNA in Huh7 and HepG2 HCC cells with higher circFOXM1 expressions, or overexpressed by transfection of circFOXM1 sequence containing plasmid pLCDH-circFOXM1 in HCCLM3 and MHCC97-H HCC cells with lower circFOXM1 expressions. As shown in Fig. 2A, circFOXM1 was effectively silenced in Huh7 and HepG2 cells and overexpressed in HCCLM3 and MHCC97-H cells determined by qRT-PCR. Then the influences of circFOXM1 genetic modification on HCC cell phenotypes were further investigated both in vitro and in vivo. The results of CCK8 showed that down-regulation of circFOXM1 expression plays an important role in reducing the proliferation of both Huh7 and HepG2 HCC cells, while increased circFOXM1 expression promoted proliferation of HCCLM3 and MHCC97-H HCC cells (Fig. 2B). Furthermore, transwell analysis showed that circFOXM1 silence effectively reduced the migration (Fig. 2C) and invasion (Fig. 2D) of Huh7 and HepG2 HCC cells, while circFOXM1 overexpression promoted the migration (Fig. 2E) and invasion (Fig. 2F) of HCCLM3 and MHCC97-H HCC cells. Moreover, the tumor volume (Fig. 2G) and the tumor weight (Fig. 2H) of the HCC xenograft produced by Huh7 cell injection were significantly inhibited by down-regulating circFOXM1 expression in Huh7 cells.

### CircFOXM1 sponges miR-1179 in HCC cells to increase SPAG5 expression

**Figure 3:**
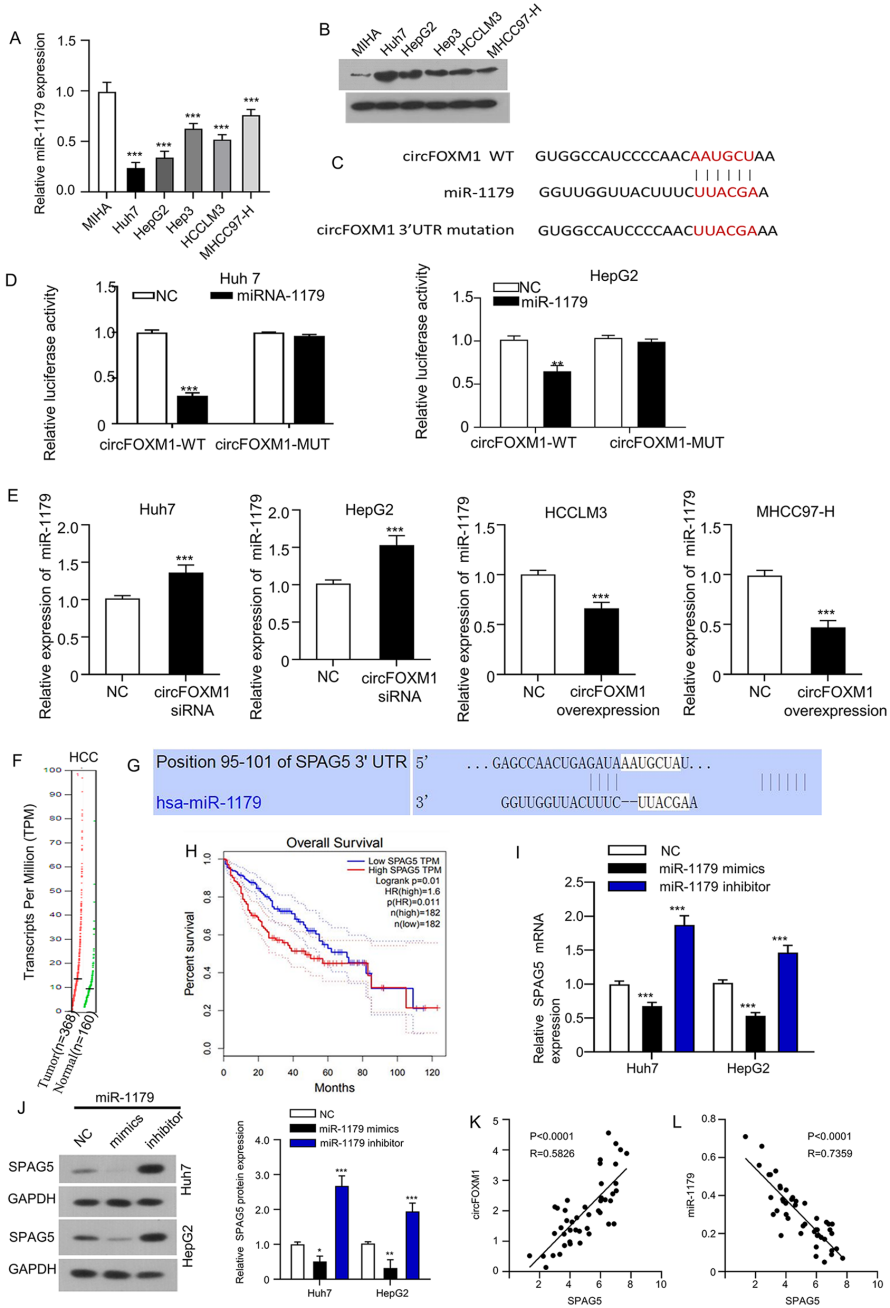
CircFOXM1 sponges miR-1179 in HCC cells to increase SPAG5 expression. (**A**) miR-1179 expression detected by qRT-PCR. (**B**) SPAG5 expression detected by Western blot assay. (**C**) Diagram of potential binding sites between miR-1179 and circFOXM1 (https://circinteractome.nia.nih.gov/) with mutation sites for specific binding assay. (**D**) dual-luciferase reporter activity of Huh7 cells co-transfected by circFOXM1 3′UTR wild type (WT) or mutated (MT) reporter with or without miR-1179 mimics; (**E**) qRT-PCR determined miR-1179 expressions in circFOXM1 silenced Huh7 and HepG2 cells, as well as in circFOXM1 overexpressed HCCLM3 and MHCC97-H cells; (**F**) SPAG5 transcripts in HCC and normal control tissues based on GEPIA database; (**G**) diagram of potential binding sites between miR-1179 with SPAG5 (http://www.targetscan.org/); (**H**) association between SPAG5 expression levels with the overall survival of HCC patients based on GEPIA database; (**I**) mRNA and (**J**) protein expressions of SPAG5 in Huh7 and HepG2 cells after miR-1179 overexpression with mimics or miR-1179 silencing with inhibitor. Pearson correlation analysis of association between circFOXM1 with SPAG5 (**K**) and miR-1179 with SPAG5 (**L**) expressions in HCC patient tissues (n = 45). *p < 0.05; **p < 0.01, ***p < 0.001.

To investigate the molecular mechanism of circFOXM1 in HCC carcinogenesis and development, miR-1179 and SPAG5 expressions were compared between HCC cells and MIHA cells, which showed that miR-1179 expression was significantly downregulated in HCC cells than in MIHA (Fig. 3A), and SPAG5 expression was significantly upregulated in HCC cells than in MIHA cells (Fig. 3B); furthermore, the potential miRNAs had complementary binding sites with circFOXM1 were first predicted using circular RNA Interactome (https://circinteractome.nia.nih.gov/) (Fig. 3C), followed by dual-luciferase reporter activity analysis in Huh7 and HepG2 HCC cells. Luciferase activities of wild-type circFOXM1 3′UTR reporter gene in Huh7 and HepG2 HCC cells were significantly inhibited when miR-1179 was overexpressed with the mimics, while the inhibited luciferase activities of circFOXM1 3′UTR reporter gene were rescued when the predicted binding sites of circFOXM1 3′UTR with miR-1179 were mutated (Fig. 3C,D). Furthermore, the direct regulation of miR-1179 by circFOXM was investigated by detecting miR-1179 expression in HCC cells after circFOXM1 was silenced or overexpressed respectively. Our results showed that miR-1179 expression was significantly upregulated in Huh7 and HepG2 HCC cells after circFOXM1 was silenced, while miR-1179 expression was significantly downregulated in HCCLM3 and MHCC97-HHCC cells after circFOXM1 was overexpressed (Fig. 3E).

To further reveal the ceRNA network triggered by circFOXM1, potential mRNAs had complementary binding sites with miR-1179 were first predicted with online tool targetscan (http://www.targetscan.org/), which showed that the 3′UTR of sperm-associated antigen 5 (SPAG5) had potential binding sites with miR-1179 (Fig. 3G). Herein, whether circFOXM1 sponges miR-1179 thus to upregulate SPAG5 expression in HCC cells was investigated. The Expression DIY module in the GEPIA database was used to analyze SPAG5 expression profile in the HCC (liver hepatocellular carcinoma, LIHC) and the normal tissues, which showed an increased trend of circFOXM1 transcripts in HCC tissues than in normal control tissues (Fig. 3F). Furthermore, the Survival Plots module in the GEPIA database was used to analyze the association between SPAG5 expression levels with the overall survival of HCC patients, which showed that HCC patients with high SPAG5 expression exhibited a significant poor overall survival (Fig. 3H). Moreover, our results showed that miR-1179 silencing with inhibitor in Huh7 and HepG2 HCC cells considerably upregulated SPAG5 expressions at both mRNA (Fig. 3I) and protein (Fig. 3J) levels, while miR-1179 overexpression with mimics in Huh7 and HepG2 HCC cells considerably downregulated SPAG5 expressions at both mRNA (Fig. 3I) and protein (Fig. 3J) levels. Pearson correlation analysis showed that circFOXM1 expression was significantly positively associated with SPAG5 expression (Fig. 3K), while miR-1179 expression was significantly negatively associated with SPAG5 expression (Fig. 3L). These findings suggested that circFOXM1 promoted HCC oncogenesis and development by sponging miR-1179 to upregulate SPAG5 expression.

### CircFOXM1/miR-1179/SPAG5 axis promotes HCC cell malignant phenotypes

**Figure 4:**
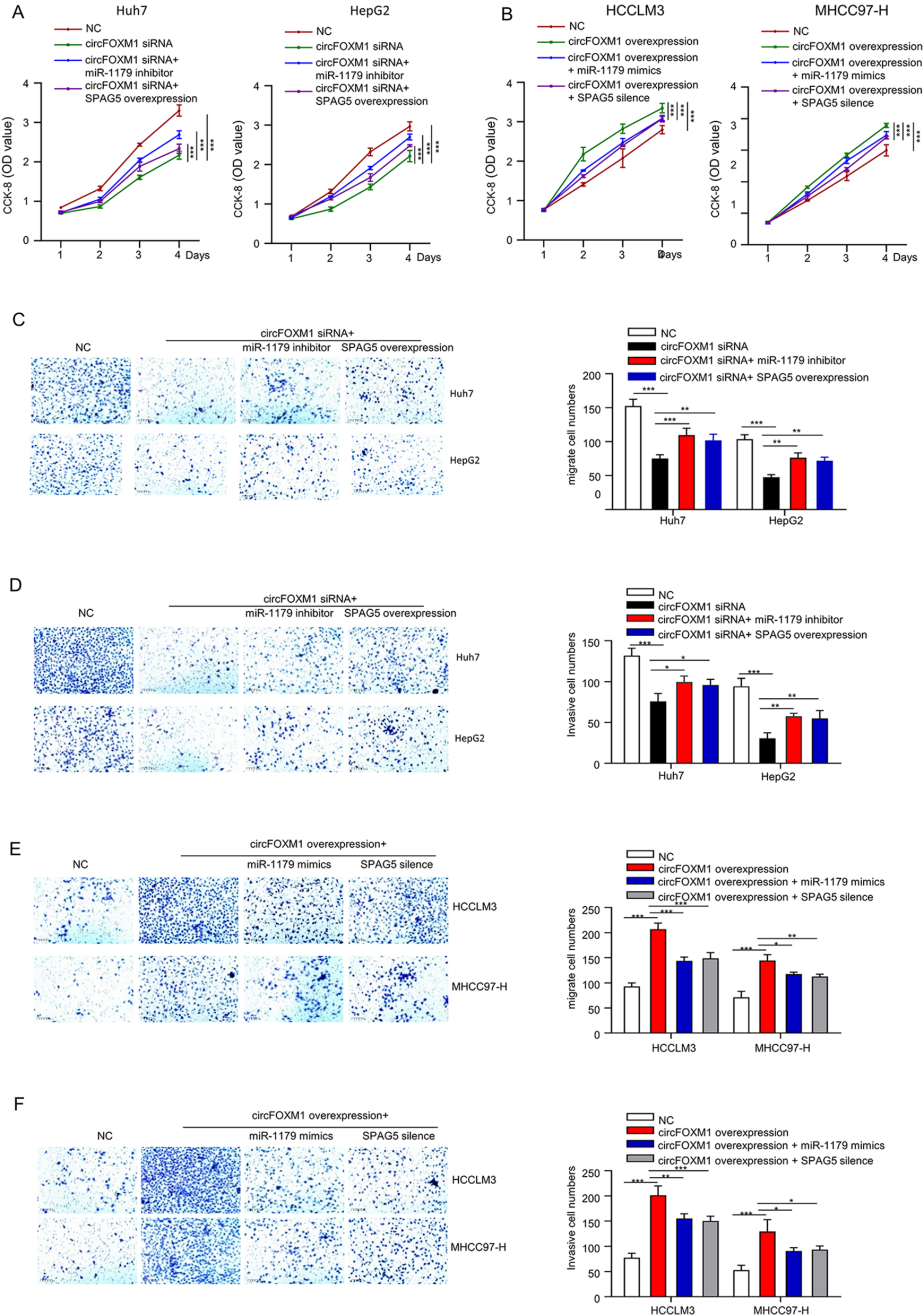
CircFOXM1 promotes viability, migration and invasion abilities of HCC cells via sponging miR-1179 to increase SPAG5 expression. (**A**) Viabilities of Huh7 and HepG2 cells after circFOXM1 silencing with/without miR-1179 inhibitor or SPAG5 overexpression determined by CCK-8 assay. (**B**) Viabilities of HCCLM3 and MHCC97-H cells after circFOXM1 overexpression with/without miR-1179 mimics or SPAG5 silencing determined by CCK-8 assay. (**C**) Migration capabilities of Huh7 and HepG2 cells after circFOXM1 silencing with/without miR-1179 inhibitor or SPAG5 overexpression determined by transwell assay without Matrigel. (**D**) Invasion capabilities of Huh7 and HepG2 cells after circFOXM1 silencing with/without miR-1179 inhibitor or SPAG5 overexpression determined by transwell assay with Matrigel. (**E**) Migration capabilities of HCCLM3 and MHCC97-H cells after circFOXM1 overexpression with/without miR-1179 mimics or SPAG5 silencing determined by transwell assay without Matrigel. (**F**) Invasion capabilities of HCCLM3 and MHCC97-H cells after circFOXM1 overexpression with/without miR-1179 mimics or SPAG5 silencing determined by transwell assay with Matrigel. *p < 0.05; **p < 0.01, ***p < 0.001.

To explore the involvement of circFOXM1/miR-1179/SPAG5 axis in HCC carcinogenesis and progression, the malignant phenotypes of HCC cells were measured following the genetic modifications. Viabilities of Huh7 and HepG2 cells after circFOXM1 silencing with/without miR-1179 inhibitor or SPAG5 overexpression were determined by CCK-8 assay. The results showed that circFOXM1 silencing inhibited OD values were partly rescued by either miR-1179 silencing or SPAG5 overexpression in a time (0–96 h) dependent manner (Fig. 4A); meanwhile, viabilities of HCCLM3 and MHCC97-H cells after circFOXM1 overexpression with/without miR-1179 mimics or SPAG5 silencing were determined by CCK-8 assay. The results showed that circFOXM1 overexpression increased OD values were partly rescued by either miR-1179 mimics or SPAG5 silencing in a time (0–96 h) dependent manner (Fig. 4B). Migration capabilities of Huh7 and HepG2 cells after circFOXM1 silencing with/without miR-1179 inhibitor or SPAG5 overexpression were determined by transwell assay without Matrigel. The results showed that circFOXM1 silencing inhibited migration capabilities were partly rescued by either miR-1179 silencing or SPAG5 overexpression (Fig. 4C). Invasion capabilities of Huh7 and HepG2 cells after circFOXM1 silencing with/without miR-1179 inhibitor or SPAG5 overexpression were determined by transwell assay with Matrigel. The results showed that circFOXM1 silencing inhibited invasion capabilities were partly rescued by either miR-1179 silencing or SPAG5 overexpression (Fig. 4D). Migration capabilities of HCCLM3 and MHCC97-H cells after circFOXM1 overexpression with/without miR-1179 mimics or SPAG5 silencing were determined by transwell assay without Matrigel. The results showed that circFOXM1 overexpression increased migration capabilities were partly rescued by either miR-1179 mimics or SPAG5 silencing (Fig. 4E). Invasion capabilities of HCCLM3 and MHCC97-H cells after circFOXM1 overexpression with/without miR-1179 mimics or SPAG5 silencing were determined by transwell assay with Matrigel. The results showed that circFOXM1 overexpression increased invasion capabilities were partly rescued by either miR-1179 mimics or SPAG5 silencing (Fig. 4F). These findings indicated that miR-1179 knockdown or SPAG5 overexpression partially reversed circFOXM1 silencing inhibited malignant phenotypes in HCC cells, meanwhile, miR-1179 overexpression or SPAG5 silencing partially reversed circFOXM1 overexpression promoted malignant phenotypes in HCC cells.

## Discussion

CircFOXM1 has been reported to contribute to sorafenib resistance of HCC cells by regulating miR-1324/MECP2^17^, indicating the potential significance of circFOXM1 in HCC management. In this study, Actinomycin D treatment verified the cyclization of circFOXM1; RNase R treatment showed that circFOXM1 was not affected by RNase R exonuclease; HCC xenograft experiment showed that circFOXM1 effectively promoted the growth of HCC cells in vivo; CCK8 assay showed that circFOXM1 effectively promoted the proliferation of HCC cells; transwell assay showed that circFOXM1 effectively promoted the migration and invasion abilities of HCC cells.

Studies have reported that SPAG5 serves a promising prognostic factor in HCC and acts as an oncogene^18–22^. Our luciferase reporter assay showed that miR-1179 could directly bind to circFOXM1 and SPAG5; miR-1179 inhibitor or SPAG5 overexpression reversed circFOXM1 siRNA-mediated inhibitory effect on HCC cell proliferation, migration and invasion abilities; and miR-1179 mimics or SPAG5 silencing reversed pLCDH-circFOXM1-mediated promotion on HCC cell proliferation, migration and invasion abilities. These findings implicate that circFOXM1 is an oncogenenic factor and plays an important role in promoting oncogenesis and metastasis of HCC.

Studies have confirmed that miR-1179 is an important tumor regulator, which directly regulates Notch signaling pathway and inhibits breast cancer cell metastasis^23^. Meanwhile, studies have also confirmed that miR-1179 can target HMGB1 to inhibit the proliferation of gastric cancer cells^24^. It has also been pointed out that miR-1179 can target the E2F5 gene in regulating pancreatic cancer cell proliferation, thereby inhibiting tumor cell migration and invasion^25^; plays an important role in regulating the proliferation of glioblastoma cells by directly targeting E2F transcription factor 5^26^; and plays important role in cancer through Akt signaling^27,28^. Moreover, circFOXM1 has been confirmed to accelerate the progression of papillary thyroid carcinoma by sponging miR-1179^10^. Literature review also revealed that SPAG5 was the downstream of miR-1179 in lung adenocarcinoma^29^.

SPAG5, also named mitotic spindle associated protein, has been reported to influence the separation of sister chromatids by regulating spindles, thus affecting the cell cycle. SPAG5 overexpression is associated with carcinogenesis and development of lung, breast, cervical and bladder urothelial cancers^22^. Therefore, the underlying mechanism of circFOXM1/miR-1179/SPAG5 axis in HCC remains to be further elucidated.

CircRNAs are derived from canonical splice sites^30–32^, with the sequences from exons, introns, antisense, intragenic, intergenic sequences or both of the exons and introns. However, the exon-derived circRNAs are the most widely studied. Regarding the biogenesis of circRNAs, multiple hypotheses have been proposed. Reverse complementary intron sequence pairing and RNA-binding protein regulation are the most commonly accepted mechanisms of biogenesis^33,34^. However, the biogenesis of circFOXM1 still need to be further explored.

In summary, our current work demonstrated that circFOXM1 promoted HCC proliferation and metastasis, which could be partially reversed by upregulating miR-1179 or downregulating SPAG5. Our results provide a theoretical basis for HCC therapy.

## Supplementary Information

Supplementary Information.

## References

[1] Bray F (2018). Global cancer statistics 2018: GLOBOCAN estimates of incidence and mortality worldwide for 36 cancers in 185 countries. CA Cancer J. Clin..

[2] Yang JD (2019). A global view of hepatocellular carcinoma: Trends, risk, prevention and management. Nat. Rev. Gastroenterol. Hepatol..

[3] Kulcheski FR, Christoff AP, Margis R (2016). Circular RNAs are miRNA sponges and can be used as a new class of biomarker. J. Biotechnol..

[4] Li X, Yang L, Chen LL (2018). The biogenesis, functions, and challenges of circular RNAs. Mol. Cell.

[5] Cortes-Lopez M, Miura P (2016). Emerging functions of circular RNAs. Yale J. Biol. Med..

[6] Cao S, Wang G, Wang J, Li C, Zhang L (2019). Hsa_circ_101280 promotes hepatocellular carcinoma by regulating miR-375/JAK2. Immunol. Cell Biol..

[7] Liu J, Liu T, Wang X, He A (2017). Circles reshaping the RNA world: From waste to treasure. Mol. Cancer.

[8] Luo Z (2020). Circular RNA circCCDC9 acts as a miR-6792-3p sponge to suppress the progression of gastric cancer through regulating CAV1 expression. Mol. Cancer.

[9] Wu Y (2019). Circular RNA circTADA2A promotes osteosarcoma progression and metastasis by sponging miR-203a-3p and regulating CREB3 expression. Mol. Cancer.

[10] Ye M, Hou H, Shen M, Dong S, Zhang T (2020). Circular RNA circFOXM1 plays a role in papillary thyroid carcinoma by sponging miR-1179 and regulating HMGB1 expression. Mol. Ther. Nucleic Acids.

[11] Lagos-Quintana M, Rauhut R, Lendeckel W, Tuschl T (2001). Identification of novel genes coding for small expressed RNAs. Science.

[12] Mollaei H, Safaralizadeh R, Rostami Z (2019). MicroRNA replacement therapy in cancer. J. Cell. Physiol..

[13] Chen K (2016). miR-490-5p suppresses tumour growth in renal cell carcinoma through targeting PIK3CA. Biol. Cell.

[14] Wu L (2018). Circ-ZNF609 promotes migration of colorectal cancer by inhibiting Gli1 expression via microRNA-150. J. BUON.

[15] Chen L (2017). circRNA_100290 plays a role in oral cancer by functioning as a sponge of the miR-29 family. Oncogene.

[16] Gao HB, Gao FZ, Chen XF (2019). MiRNA-1179 suppresses the metastasis of hepatocellular carcinoma by interacting with ZEB2. Eur. Rev. Med. Pharmacol. Sci..

[17] Weng H (2021). circFOXM1 contributes to sorafenib resistance of hepatocellular carcinoma cells by regulating MECP2 via miR-1324. Mol. Ther. Nucleic Acids.

[18] Yang YF (2018). SPAG5 interacts with CEP55 and exerts oncogenic activities via PI3K/AKT pathway in hepatocellular carcinoma. Mol. Cancer.

[19] Zhou H, Wang SC, Ma JM, Yu LQ, Jing JS (2018). Sperm-associated antigen 5 expression is increased in hepatocellular carcinoma and indicates poor prognosis. Med. Sci. Monit..

[20] Liu H (2018). SPAG5 promotes hepatocellular carcinoma progression by downregulating SCARA5 through modifying beta-catenin degradation. J. Exp. Clin. Cancer Res..

[21] Roy S (2018). microRNA 193a–5p regulates levels of nucleolar- and spindle-associated protein 1 to suppress hepatocarcinogenesis. Gastroenterology.

[22] Chen W, Chen X, Li S, Ren B (2020). Expression, immune infiltration and clinical significance of SPAG5 in hepatocellular carcinoma: A gene expression-based study. J. Gene Med..

[23] Li WJ (2018). Increased expression of miR-1179 inhibits breast cancer cell metastasis by modulating Notch signaling pathway and correlates with favorable prognosis. Eur. Rev. Med. Pharmacol. Sci..

[24] Li Y, Qin C (2019). MiR-1179 inhibits the proliferation of gastric cancer cells by targeting HMGB1. Hum. Cell.

[25] Lin C (2018). MicroRNA-1179 inhibits the proliferation, migration and invasion of human pancreatic cancer cells by targeting E2F5. Chem. Biol. Interact..

[26] Xu X (2017). MicroRNA-1179 inhibits glioblastoma cell proliferation and cell cycle progression via directly targeting E2F transcription factor 5. Am. J. Cancer Res..

[27] Song L (2018). MicroRNA-1179 suppresses cell growth and invasion by targeting sperm-associated antigen 5-mediated Akt signaling in human non-small cell lung cancer. Biochem. Biophys. Res. Commun..

[28] Gao Y, Xu H, Pu T (2020). MicroRNA-1179 suppresses the proliferation and enhances vincristine sensitivity of oral cancer cells via induction of apoptosis and modulation of MEK/ERK and PI3K/AKT signalling pathways. AMB Express.

[29] Wang L (2020). LncRNA LINC00857 regulates lung adenocarcinoma progression, apoptosis and glycolysis by targeting miR-1179/SPAG5 axis. Hum. Cell.

[30] Jeck WR (2013). Circular RNAs are abundant, conserved, and associated with ALU repeats. RNA.

[31] Memczak S (2013). Circular RNAs are a large class of animal RNAs with regulatory potency. Nature.

[32] Salzman J, Gawad C, Wang PL, Lacayo N, Brown PO (2012). Circular RNAs are the predominant transcript isoform from hundreds of human genes in diverse cell types. PLoS One.

[33] Zhang XO (2014). Complementary sequence-mediated exon circularization. Cell.

[34] Conn SJ (2015). The RNA binding protein quaking regulates formation of circRNAs. Cell.

