# Supplementary figures and images for: CircFOXM1 promotes proliferation and metastasis of hepatocellular carcinoma via regulating miR-1179/SPAG5 axis
Source: Sci Rep. 2021 Dec 13;11:23890. doi: 10.1038/s41598-021-03285-w (PMC8668908; doi:10.1038/s41598-021-03285-w)

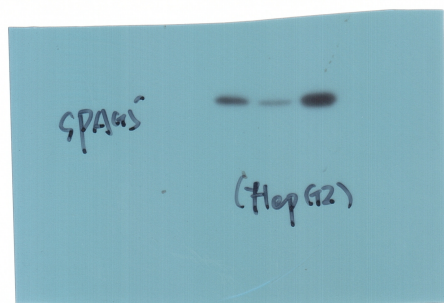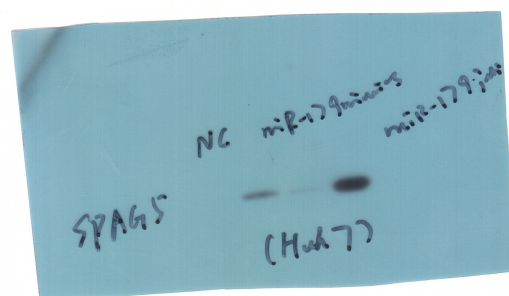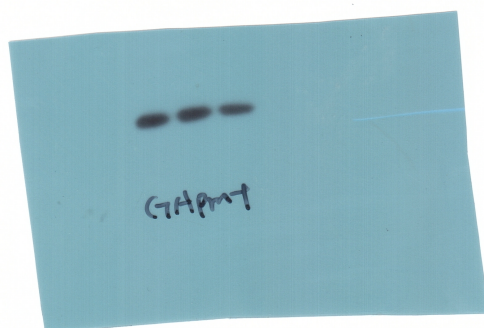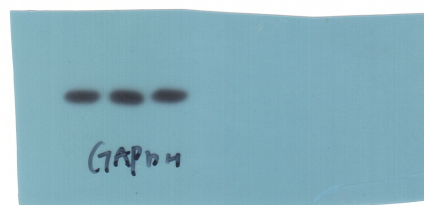

Supplement: Supplementary file 1 — Supplementary Information. [file 41598_2021_3285_MOESM1_ESM.pdf]
